# Effects of a hypomagnetic field on DNA methylation during the differentiation of embryonic stem cells

**DOI:** 10.1038/s41598-018-37372-2

**Published:** 2019-02-04

**Authors:** Soonbong Baek, Hwan Choi, Hanseul Park, Byunguk Cho, Siyoung Kim, Jongpil Kim

**Affiliations:** 1 Department of Biomedical Engineering, Dongguk University, Seoul, 100-715 South Korea; 2 Department of Chemistry, Dongguk University, Seoul, 100-715 South Korea

## Abstract

It has been reported that hypomagnetic fields (HMFs) have a negative influence on mammalian physiological functions. We previously reported that HMFs were detrimental to cell fate changes during reprogramming into pluripotency. These studies led us to investigate whether HMFs affect cell fate determination during direct differentiation. Here, we found that an HMF environment attenuates differentiation capacity and is detrimental to cell fate changes during the *in vitro* differentiation of embryonic stem cells (ESCs). Moreover, HMF conditions cause abnormal DNA methylation through the dysregulation of DNA methyltransferase3b (Dnmt3b) expression, eventually resulting in incomplete DNA methylation during differentiation. Taken together, these results suggest that an appropriate electromagnetic field (EMF) environment may be essential for favorable epigenetic remodeling during cell fate determination via differentiation.

## Introduction

Recently, there has been increasing public interest in the biological effects of electromagnetic fields (EMFs). In particular, it is reported that EMFs with frequencies ranging from 1 to 300 hertz (Hz) affect various cellular properties such as proliferation^1,2^, migration^3,4^, apoptosis^5,6^ and stress responses^7,8^. Moreover, EMFs are involved in lineage commitment during cell fate conversion. For example, EMF exposure significantly promotes the neural differentiation of embryonic neural stem cells (NSCs) through activation of the transient receptor potential canonical1 (TRPC1)^9^. Furthermore, it has been reported that a specific EMF frequency enhances the osteogenic differentiation of bone marrow stem cells (BMSCs)^10^. In addition, EMF exposure to mesenchymal stem cells (MSCs) induces the epidermal growth factor signaling cascade, which facilitates neuronal gene expression^11^. Taken together, these studies collectively indicate that EMFs regulate cell fate conversion.

Although several studies have reported direct evidence of the effects of EMFs on cell fate plasticity, hypomagnetic fields (HMFs, <5000 nT) also substantially affect cellular metabolism^12^, the morphology of organisms^13^ and the development of embryos^14^. For example, HMFs accelerate the proliferation of human neuroblastoma cells by disturbing the G1/S transition^15^. HMFs also affect mitochondrial function in mouse skeletal muscle cells^16^. Moreover, a recent study presented significant evidence that HMFs regulate actin assembly, in cells and subsequently affect cell motility^17^.

Previously, we and others have shown that an HMF environment causes epigenetic changes during cell fate determination^18^. In our study, we reported that exposure to an EMF induced epigenetic changes during cell reprogramming by mediating histone modification, whereas an HMF environment was detrimental to cell fate changes in epigenetic reprogramming, suggesting that an HMF environment is critical for epigenetic changes during cell fate conversion. Thus, these results raise the interesting possibility of a fundamental role for HMF conditions in establishing cellular identity. To address this hypothesis, in the present study, we investigated whether an HMF environment influences the differentiation of embryonic stem cells (ESCs) and identified the underlying mechanisms that mediate the effects of an HMF on cell differentiation.

We found that an HMF environment inhibits the differentiation of ESCs, accompanied by abnormal DNA methylation. Moreover, the HMF-induced phenotypes observed during ESC differentiation were significantly rescued by the introduction of Dnmt3b, suggesting that the lack of an EMF causes abnormal DNA methylation during ESC differentiation. These results suggest that HMF environments influence epigenetic modifications that drive ESC cell fate changes. Thus, our study provides a foundation for understanding the effects of EMFs on biological processes such as cell fate determination.

## Results

### Early differentiation of mESCs in an HMF environment

**Figure 1:**
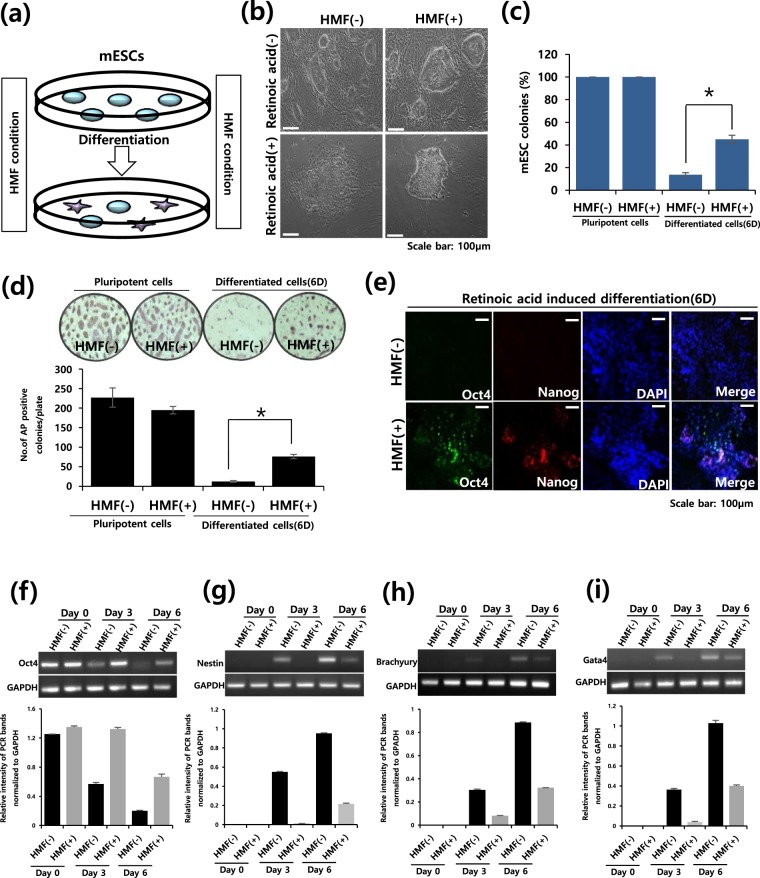
Aberrant mESC differentiation in an HMF. (**a**) Schematic drawing of HMF exposure during mESC differentiation. (**b**) Morphology of RA-treated mESCs under normal (HMF(−)) and HMF(+) condition. Experiment were performed in triplicate. (**c**) Percentage of mESC colonies after RA induced differentiation. Experiments were performed in triplicate. (**d**) Representative image (top) and a number of AP-positive colonies (bottom) of RA-treated mESCs under normal and HMF conditions, normalized. Data represent the mean ± SEM. Student’s t-test, *P < *0*.*05* (*n* = 3). (**e**) Immunostaining of mESCs at 6 days after RA treatment for Oct4 and Nanog. Experiment were performed in triplicate. (**f**–**i**) Semi-quantitative RT-PCR representative band images (top) and relative intensity (bottom) of the pluripotency gene (Oct4) and markers for three germ layers (Pax6, Brachyury and Gata4), normalized to GAPDH (Full-length gel image was included in Supplementary Fig. 6a).

To investigate the effects of HMF conditions on early differentiation, we induced the differentiation of mESCs with retinoic acid (RA) for six days under HMF conditions using a three-axis Helmholtz coil (Supplementary Figs 1a and 1a). mESCs under normal conditions showed a differentiated morphology six days after RA treatment, whereas the majority of mESC colonies under HMF conditions maintained an undifferentiated morphology (Fig. 1b,c). Additionally, more alkaline phosphatase (AP)-positive colonies were observed under HMF conditions than in the control group (Fig. 1d). Consistent with these results, mESCs exposed to HMF conditions showed residual expression of Oct4 and Nanog, known as markers of pluripotency, unlike the control group, as confirmed by immunostaining (Fig. 1e). We also observed that HMF conditions inhibited the expression of markers corresponding to the pluripotent and three germ layers, including Oct4, Nestin (ectoderm), Brachyury (mesoderm) and Gata4 during mESC differentiation (Fig. 1f–i). Additionally, the long-term differentiation of mESCs up to 30 days under HMF conditions resulted in the failure of proper differentiation (Supplementary Fig. 1b). We observed lower expression of three germ layer markers such as Tuj1, Brachyury and Foxa2 (Supplementary Fig. 1b), whereas higher expression of pluripotency genes such as Oct4 and Nanog at 30 days of differentiation (Supplementary Fig. 1c). Moreover, we confirmed upregulation of Nestin, Brachyury and Gata4 in the HMF-treated cells after withdrawal of HMF conditions, suggesting that the detrimental effects on mESC differentiation by HMF condition were partially rescued under normal conditions (Supplementary Fig. 1d). However, HMF conditions did not affect the capacity of mESCs gene expression (Supplementary Fig. 2a–c) or proliferation during the process of differentiation (Supplementary Fig. 2d). Thus, these results suggest that the HMF environment markedly delayed the early differentiation of mESCs.

### Neuronal differentiation of mESCs in the HMF environment

**Figure 2:**
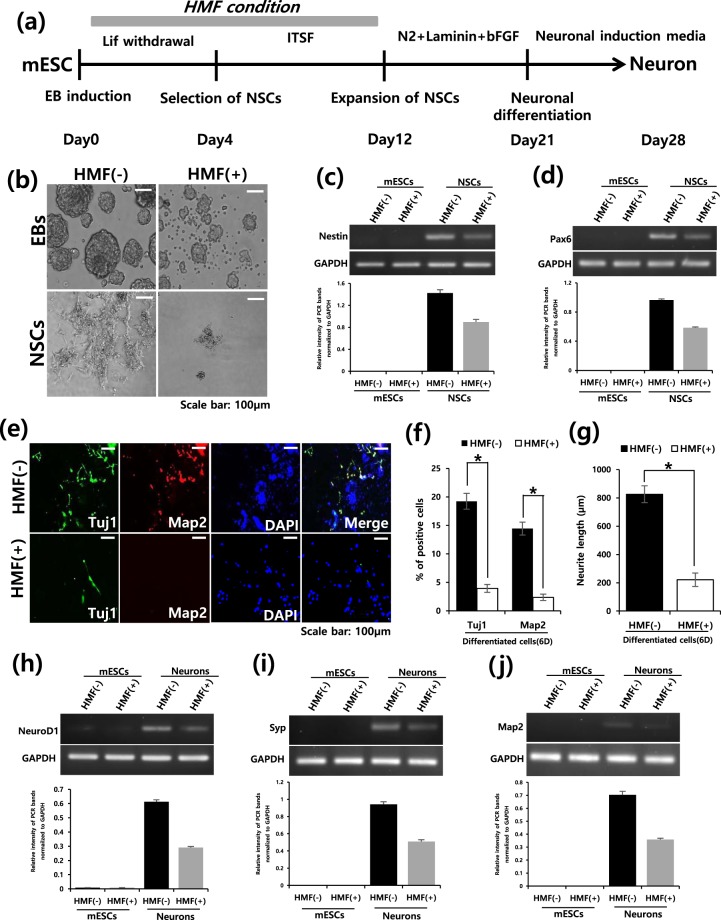
HMF suppresses the neuronal differentiation of mESCs. (**a**) Schematic diagram describing EB-based neuronal differentiation. (**b**) Representative images of EB formation under normal (HMF(−)), HMF(+) condition at day 12. Experiments were performed in triplicate. (**c**,**d**) Representative band images (top) and relative intensity (bottom) of semi-quantitative RT-PCR analysis for NSC markers (Nestin and Pax6) 12 days after neuronal differentiation, normalized to GAPDH. (Full-length gel image was included in Supplementary Fig. 6a). (**e**) Immunofluorescence staining for neuronal markers (Tuj1 and Map2) of differentiated neurons under normal (HMF(−)) and HMF(+) condition. Experiment were performed in triplicate. (**f**) Quantification of Tuj1/Map2 positive cells. Data represent the mean ± SEM. Student’s t-test, *P < *0*.*05* (*n* = 3). (**g**) Graph of average measured neurite lengths 28 days after neuronal differentiation under normal and HMF condition. Data represent the mean ± SEM. Student’s t-test, *P < *0*.*05* (*n* = 3). (**h**–**j**) Semi-quantitative RT-PCR analysis band images (top) and relative intensity (bottom) of neuronal genes (NeuN, Syp and Map2) in differentiated neurons, normalized to GAPDH (Full-length gel image was included in Supplementary Fig. 6a).

To better understand the effects of HMF on differentiation, we further investigated whether HMF conditions influence terminal differentiation. mESCs were induced to neuronal lineages by forming embryoid bodies (EBs) (Fig. 2a). Interestingly, we found that neuronal differentiation via EB formation under HMF conditions did not result in the formation of proper EBs, in contrast to the EBs observed under normal conditions (Fig. 2b). This decrease of EB formation under HMF conditions could be rescued by HMF withdrawal (Supplementary Fig. 3a,b), suggesting impaired EB formation is HMF condition dependent. Consistent with this result, further EB based neural differentiation in the HMF environment resulted in a substantial reduction in the expression of neural stem cell (NSC) marker genes such as Nestin and Pax6 (Fig. 2c,d). The percentages of tubulin-beta3 (Tuj1)- and microtubule associated protein2 (MAP2)-positive cells were substantially reduced by the HMF environment (Fig. 2e,f). In addition, the neurite length of differentiated NSCs was considerably decreased in the HMF-treated group (Fig. 2g). Moreover, during terminal differentiation under HMF exposure, we observed reduced mRNA levels of the following neuronal markers: Neuronal differentiation1 (NeuroD1), Synaptophysin (Syp) and Microtubule associated protein2 (Map2) (Fig. 2h–j). These findings show that HMF conditions inhibit or delay neuronal differentiation, suggesting that the HMF environment has detrimental effects on cell fate conversion during mESC differentiation.

### Effects of HMF on DNA methylation during differentiation

**Figure 3:**
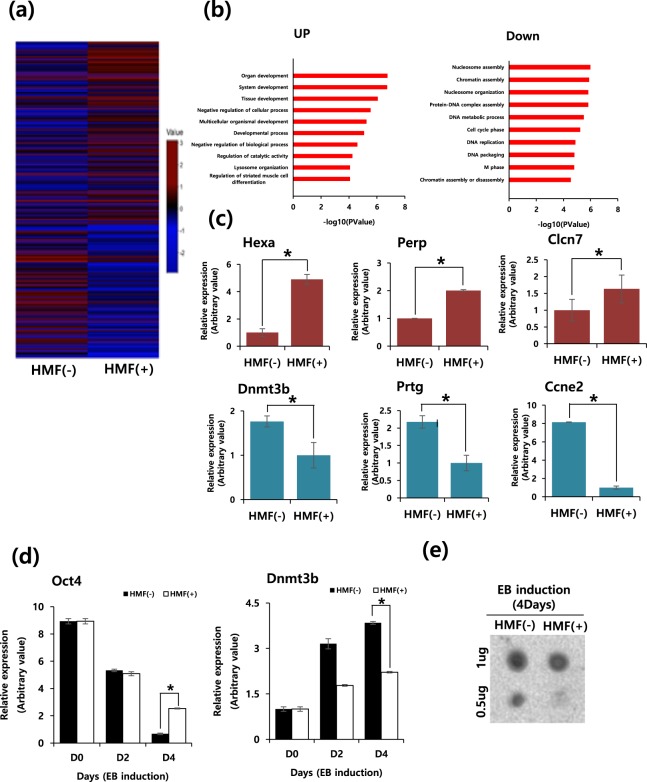
Effects of HMF on DNA methylation during mESC differentiation. (**a**) Global gene expression profiling of mESCs at 2 days after EB induction in Normal (HMF(−)) and HMF(+) condition. (**b**) Subsets of upregulated (left) and downregulated (right) genes determined by GO term analysis in HMF(+) condition. (**c**) qRT-PCR validation of the microarray analysis of upregulated (Hexa, Perp and Clcn7)) and downregulated genes (Dnmt3b, Prtg and Ccne2). Values are normalized to GAPDH mRNA expression. Results are displayed as arbitrary value (expression in control = 1) Data represent the mean ± SEM. Student’s t-test, *P < *0*.*05* (*n* = 3). (**d**) Time course of Oct4 and Dnmt3b mRNA expression during mESC differentiation, normalized to GAPDH. Experiments were performed in triplicate. (**e**) Dot blot assay using an anti-5mC antibody at 2 days after EB induction. Experiments were performed in triplicate. Blot was prepared from same gel and photographed with the Bio-Rad Gel Doc system (Full-length gel image was included in Supplementary Fig. 7a).

In order to understand the underlying molecular mechanisms of HMF condition in the differentiation, we investigated the global gene expression profile during early mESC differentiation using a DNA microarray (Fig. 3a). Since the HMF condition dramatically delayed the differentiation of mESCs (Fig. 1d), the microarray analysis was done at day 2 EBs to avoid significant phenotypic changes in the control and HMF treated groups (Supplementary Fig. 4a).

Remarkably, HMF exposure resulted in dramatic changes in global gene expression during mESC differentiation (Fig. 3a,b). We identified 983 genes whose expression was significantly changed (≥2-fold) after HMF exposure and gene ontology (GO) term analysis across two independent experimental sets showed differentially expressed gene signatures associated with developmental processes in the HMF-treated group (Fig. 3b and Supplementary Table 1). Moreover, we found that several negative transcription regulators including Ahnak2, Ndrg1 and Grn were upregulated under the HMF (+) conditions (Supplementary Fig. 4b), which might have been responsible for the negative effects on differentiation of mESCs under HMF condition. We observed that the most heavily downregulated genes were DNA methyltransferase 3b (DNMT3b), protogenin (Prtg), and cyclin E2 (Ccne2) (Fig. 3c and Supplementary Table 2).

In particular, we found an epigenetic modifier, Dnmt3b, that was significantly downregulated during ESC differentiation under HMF conditions. We also found no effect on the expression of other Dnmt3 family members such as Dnmt3A and Dnmt3l in mESC differentiation under HMF conditions (Supplementary Fig. 4c). This regulation was confirmed using quantitative real-time PCR (Fig. 3c). Dnmt3b mRNA levels were nearly two-fold lower during HMF differentiation than in the control group, whereas Oct4 mRNA levels poorly decreased under HMF conditions (Fig. 3d), potentially because HMF leads to delayed differentiation via insufficient DNA methylation^19^. To further investigate whether the change in Dnmt3b activity under HMF conditions directly influences the genomic distribution of 5mC in the genome, we performed a dot blot assay four days after mESCs differentiation (Fig. 3e). There was a much greater decrease in genomic 5mC levels during the differentiation of mESCs exposed to HMF (Fig. 3e). Bisulfite sequencing confirm that *de novo* methylation to Oct4 and Nanog promoter is impaired during mESCs differentiation under HMF condition (Supplementary Fig. 4d). These data showed that the HMF environment decisively influences the methylation of gene expression via Dnmt3b downregulation during the ESC differentiation process.

### Overexpression of Dnmt3b rescues HMF phenotypes

**Figure 4:**
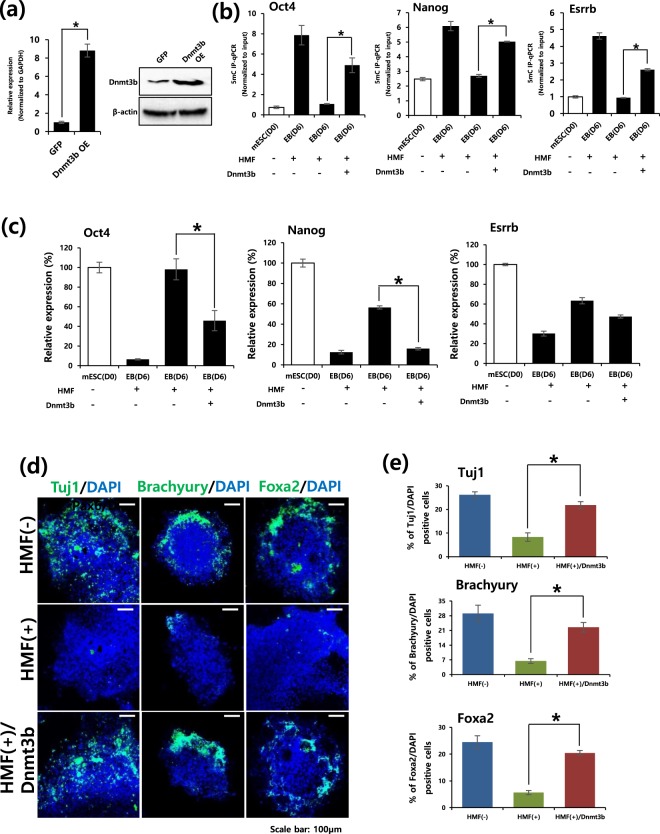
Overexpression of Dnmt3b rescues HMF phenotypes during mESC differentiation. (**a**) Confirmation of Dnmt3b mRNA levels by (left) qRT-PCR and (right) western blot after Dnmt3b overexpression in mESC differentiation. qRT-PCR data represent the mean ± SEM. Student’s t-test, *P < *0*.*05*, normalized to GAPDH (*n* = 3). (Full-length gel image was included in Supplementary Fig. 7b). (**b**) IP-qRT-PCR for 5mC at Oct4, Nanog and Esrrb promoter locus after 3 days of EB induction, normalized to input (3 days after ESC differentiation). Data represent the mean ± SEM. Student’s t-test, *P < *0*.*05* (*n* = 3). (**c**) qRT-PCR analysis to measure Oct4, Nanog and Esrrb gene expression during mESC differentiation, normalized to GAPDH. Data represent the mean ± SEM. Student’s t-test, *P < *0*.*05* (*n* = 3). (**d**) Immunostaining to confirm the expression of markers corresponding to the three germ layers at 3 days after EB plating (7 days after ESC differentiation). Experiments were performed in triplicate. (**e**) Quantification of Tuj1- (left), Brachyury- (middle) and Foxa2-positive (right) cells. Data represent the mean ± SEM. Student’s t-test, *P < *0*.*05* (*n* = 3).

Next, to demonstrate that HMF conditions are truly responsible for incomplete DNA methylation during mESC differentiation, we overexpressed Dnmt3b during HMF-exposed differentiation (Fig. 4a). We confirmed that exogenous Dnmt3b mRNA levels in mESCs increased with the introduction of a Dnmt3b-overexpressing lentivirus (Fig. 4a). After the differentiation of mESCs, we determined the amount of 5mC at the promoter of the pluripotency genes Oct4, Nanog, and Esrrb to examine the effects of HMF conditions on methylation levels of these genes (Fig. 4b). Consistent with previous results, HMF conditions significantly induced a decrease in 5mC levels at the promoter loci of pluripotency genes, whereas Dnmt3b overexpression during ESC differentiation was found to increase methylation at the promoters of pluripotency genes, based on the observed increase in 5mC levels (Fig. 4b). These results demonstrate that HMF conditions affect global DNA methylation. Additionally, we confirmed that no dramatic changes of DNA methylation occurred during ESC culture under HMF condition (Supplementary Fig. 5a). Moreover, the expression level of the pluripotency genes Oct4, Nanog, and Esrrb was more efficiently reduced due to the overexpression of Dnmt3b during HMF-exposed differentiation than during HMF-exposed differentiation without Dnmt3b overexpression (Fig. 4c).

Moreover, Oct4, Nanog and Esrrb levels were more efficiently reduced by the overexpression of Dnmt3b during HMF-exposed differentiation than during HMF-exposed differentiation alone (Fig. 4c). We confirmed the above findings in further differentiation via immunostaining to detect markers corresponding to the three germ layer markers Tuj1 (ectoderm), Brachyury (mesoderm), and Foxa2 (endoderm) (Fig. 4d). Consistent with previous results, Dnmt3b overexpression at seven days after ESC differentiation led to an increase in expression of the three germ layer markers Tuj1, Brachyury, and Foxa2 (Fig. 4d,e and Supplementary Table 3). Taken together, these results suggest that the detrimental effects of HMF exposure on ESC differentiation were efficiently rescued by the overexpression of Dnmt3b, indicating that abnormal DNA methylation mediates the detrimental effects of HMF exposure during ESC differentiation.

## Discussion

Several studies have reported that HMF, a very weak magnetic field environment, affects diverse physiological mechanisms^20,21^ and the morphology of organisms^22,23^. In the present study, we focused on the effects of HMF on the differentiation of ESCs and examined whether an HMF environment affects epigenetic changes during cell differentiation. Previous studies demonstrated that an HMF environment was detrimental to cell fate changes during epigenetic reprogramming^18^. In this study, we show that environmental EMF conditions are critical for the rapid and precise response of epigenetic changes to environmental signals during cell fate determination. It is not clear why EMF exposure selectively affects cells undergoing dynamic epigenetic changes, but one can speculate that active chromatin remodeling is susceptible to the effects of EMFs, while cells with a stable identity under constant culture conditions exhibit less active chromatin dynamics, rendering these cells largely resistant to the effects of EMFs. Consistent with this idea, we found that HMF conditions delayed cell fate conversion during the differentiation of mESCs, whereas there were no effects on ESCs in a pluripotent state. In addition, reduced expression of markers corresponding to the three germ layers during ESC differentiation and reduced EB formation under HMF conditions indicate that an EMF is critical for the normal differentiation of mESCs.

Many studies have demonstrated that cell differentiation occurs through the methylation of genomic DNA in cells^24^. The Oct4 gene is a target gene controlled by DNA methylation in pluripotent stem cells, and aberrant Oct4 gene expression inhibits cell differentiation^25^. A reduction in Oct4 gene expression leads to the differentiation of ESCs^26^ and is strongly related to the activity of DNA methyltransferase^27^. In accordance with these results, Oct4 gene expression was gradually reduced under HMF conditions, whereas Dnmt3b activity was not induced during differentiation. Based on these results, we presume that the delayed differentiation of mESCs under HMF conditions may be decisively influenced by the reduced activity of Dnmt3b. As an epigenetic mechanism for DNA modification, DNA methylation involves DNA methyltransferases such as Dnmt3b^28^. Previous evidence demonstrated that Dnmt3b is related to epigenetic reprogramming and is commonly expressed in germline^29^ and pluripotent stem cells. In light of our results, we conclude that Dnmt3b contributes to the alteration of genomic DNA methylation patterns in mESCs in an HMF environment. However, previous reports regarding individual knockouts of Dnmt3 in mouse and human ESCs did not inhibit their differentiation abilities^30–32^, indicating that Dnmt3b was not fully responsible for mediating effects of the HMF (+) conditions. At the minimum, we can report that DNA methylation was involved in HMF-mediated detrimental effects, and overexpression of Dnmt3b partially rescued these phenotypes. Future studies investigating the detailed mechanisms by which Dnmt3b senses HMFs will provide more insight into the relationship between HMFs and epigenetic plasticity. Considering our results, we conclude that EMFs play a role in establishing cellular identity, and it will be highly interesting to apply EMFs in specific therapeutic paradigms for which epigenetic reorganization is a critical outcome.

## Experimental Section

### Mouse embryonic stem cells (mESCs) culture

mESCs (V6.5) were grown on a mitotically inactivated mouse embryonic fibroblast (MEF) feeder layer in DMEM (GIBCO, Grand Island, NY, USA) supplemented with 10% fetal calf serum (HyClone, Logan, UT), 1 mM nonessential amino acids (Millipore, Temecula, CA, USA), 0.1 mM 2-mercaptoethanol (Millipore, Temecula, CA, USA), 1% L-glutamine (Millipore, Temecula, CA, USA), 1% penicillin/streptomycin (GIBCO, Grand Island, NY, USA), and 0.1% leukemia inhibitory factor (ESGRO, Darmstadt, Germany) at 37 °C in a humidified atmosphere with 5% CO_2_.

### Generation of an HMF for cell exposure

A system for the generation of three-dimensional (3D) HMF conditions was previously reported. Briefly, to generate a zero-field environment by canceling the earth’s magnetic field in a cell culture incubator, we used three-axis Helmholtz coils within a three-axis magnetic sensor (DC Milligauss Meter 3 axis, AlphaLab Inc., USA). A 3D EMF generator created reverse magnetic fields to maintain a magnetic field-free space by adjusting the voltage across the coil with a power supply (PS2520G, Tektronix, USA). The cell cultures were located in the center of the three-axis Helmholtz coil in the incubator, and control cells were grown in a different CO_2_ incubator under the same conditions, without the Helmholtz coil.

### Differentiation of mESCs into neurons

mESC colonies were harvested using 0.125% trypsin, separated from the MEF feeder cells by gravity, gently triturated and cultured for 4 days in nonadherent suspension culture dishes in Embryoid body (EB) medium (DMEM containing 15% FBS (HyClone, Logan, UT), 1% nonessential amino acids (GIBCO, Grand Island, NY, USA) and 0.1 mM ß-mercaptoethanol (GIBCO, Grand Island, NY, USA). Subsequently, EBs were plated onto adherent tissue culture dishes in ITSF (DMEM/F12 (GIBCO, Grand Island, NY, USA) with 6.25 mg/ml insulin (Corning, USA)), 25 mg/ml transferrin (Sigma, USA), 0.5 mM selenium chloride (Sigma, USA), 2.5 mg/ml fibronectin (Sigma, USA), and 1% penicillin/streptomycin (GIBCO, Grand Island, NY, USA). After 8 days of selection for neural precursors, cells were dissociated using 0.125% trypsin/EDTA (GIBCO, Grand Island, NY, USA) and plated on a 15 μg/ml poly-L-ornithine (Sigma, USA)-coated culture dish in N3 medium^33^.

### DNA immunoprecipitation (IP) with an anti-5-methylcystosine (anti-5mC) antibody

gDNA was isolated using GeneJet Genomic DNA Purification kit (Thermo Scientific, USA). gRNA fragmentation was performed by NEBNext dsDNA fragmentase (NEB, England). 5 ug mono-clonal 5mC antibody (Millpore, Temecula, CA, USA) were incubated for 4 h with 5 μg fragmented gDNA at 4 °C with rotation. 40 ul of Dynabeads A and G were pre-washed three times with 1 ml 0.1% PBS–BSA, respectively. Beads were resuspended in 500 μl IP buffer (100 mM Na-phosphate pH 7.0, 0.14 M NaCl and 0.05% Triton X-100) added to the DNA–antibody mixtures, and incubated for 2overnight at 4 °C with rotation. The Bead–DNA-antibody mixture was washed three times with 500 μl IP buffer. Beads were resuspended in 500 μl digestion buffer (50 mM Tris pH 8.0, 10 mM EDTA and 0.5% SDS and 5 μl proteinase K (10 mg/ml; Sangon)), then incubated for 2 h at 60 °C. DNA was purified using Chromatin IP DNA Purification Kit (Active Motif, Carlsbad, CA). The primer sequence for Oct4, Nanog and Esrrb promoter to amplify the promoter of CpG is–lands approximately −1 to +500 bp region are listed in Supplementary Table 4.

### Quantitative RT-PCR Analysis

Total RNA was purified using an eCube Tissue RNA Mini Kit (PhileKorea, Korea). Briefly, 500 ng of DNase-treated RNA was reverse-transcribed using a First Strand Synthesis Kit (Bioneer, Korea). Quantitative RT-PCR analysis was performed in triplicate using a Rotor-Gene Q Real-Time PCR System (Qiagen, Germany) with a SYBR Fast qPCR Kit (KAPA Biosystems, USA). The value was normalized by GAPDH mRNA expression. Primer sequences used in this study are listed in Supplementary Table 4.

### Semi-quantitative RT-PCR

Semi-quantitative RT-PCR was performed using the Applied Biosystem Veriti Thermal Cycler. Reaction conditions were 1 cycle of 94 °C for 5 minutes, followed by 40 cycles of 30 seconds at 94 °C, 40 seconds at 58 °C and 15 seconds at 72 °C. Semi-quantitative RT-PCR carried out in 20 μL volumes using 10 μL of 2x the Accupower® PCR premix (Bioneer, Korea), 10 pmol/μL each primer set and 30 ng cDNA per reaction. Primer sequences used in this study are listed in Supplementary Table 4.

### Immunofluorescence

After fixation for 20 min with 4% paraformaldehyde at room temperature, mESCs were permeabilized with 0.0125% Triton X-100. Then, the mESCs were incubated with 1% BSA and incubated overnight with a primary antibody specific to Tuj1 (1:500; Sigma, St. Louis, MO, USA), Map2 (1:500; Cell Signaling Technology, USA), Brachyury (1:500; Santa Cruz, CA, USA), Foxa2 (1:500; Santa Cruz, CA, USA), Oct4 (1:500; Santa Cruz, CA, USA), or Nanog (1:500; Bethyl Lab, Montgomery, TX, USA). After washing with PBS, mESCs were incubated at room temperature for 90 min with specific secondary antibodies (1:1000; Invitrogen, Carlsbad, CA, USA). Counterstaining was performed with DAPI (5 mg/ml, Sigma, St. Louis, MO, USA). Cells ere imaged with a Nikon eclipse Ti. The three-color images were saved as a tif file format and merged with Adobe Photoshop software.

### Microarray processing and hybridization

2 days after EB induction in normal and HMF conditions, we purified RNA using Tissue RNA Mini Kit (PhileKorea, Korea). The Affymetrix Whole transcript Expression array process was executed according to the manufacturer’s protocol (GeneChip Whole Transcript PLUS reagent Kit). cDNA was synthesized using the GeneChip WT (Whole Transcript) Amplification kit as described by the manufacturer. The sense cDNA was then fragmented and biotin-labeled with TdT (terminal deoxynucleotidyl transferase) using the GeneChip WT Terminal labeling kit. Approximately 5.5 μg of labeled DNA target was hybridized to the Affymetrix GeneChip Mouse 2.0 ST Array at 45 °C for 16 hour. Hybridized arrays were washed and stained on a GeneChip Fluidics Station 450 and scanned on a GCS3000 Scanner (Affymetrix).

### Statistical analysis of microarray data

Background subtraction and normalization using the robust multi array average algorithm (RMA) method was performed by GeneSpring GX 11.5 software (Agilent Technologies) for gene expression experiments. Fold change values for genes were calculated as the ratio of the signal values of the experimental (TM or ALLN treated) group compared with the control group. Gene expression changes with >2-fold alterations were considered significant.

### Bisulfite sequencing

Bisulfite sequencing was performed according to the manufacture’s instruction (EpiJET Bisulfite conversion kit, Thermo scinetific). 20 ~ 50 ng of bisulfite-treated DNA was used I n a standard PCR protocol to amplify Oct4 and Nanog promoter region in mouse V6.5 mESCs, EB and HMF treated EB, PCR producets were cloned into the pCR2.1 vector (Invitrogen) and sequenced using the M13 primer. Oct4 primer F: gaacagttttgccaagctgctg R: ccggttacagaaccatactcg Nanog primer F: aactcacataccagatgggctt R: aagcataatgggctcagacac.

### Lentiviral transduction of Dnmt3b

Construction of lentiviral vectors containing Dnmt3b or GFP under a minimal CMV promoter was generated after EcoRI cloning from a FUW lentivirus backbone. Lentiviruses were produced using a standard calcium phosphate transfection protocol (Promega, Madison WI). For lentiviral transfection, 293T cells were maintained in complete culture medium at 37 °C and 5% CO2 at 50-70% confluence in T25 flasks. Dnmt3b and GFP lentiviral vector were combined with expression (pR8DE) and packaging (VSV-G) vectors and used to transfect 293T cells using the calcium phosphate transfection protocol. Viral supernatant was collected and filtered through 0.45 μm filter 48 hr later, and immediately used to transduce recipient cells. mESCs (v6.5) were transduced (40,000 cells) at passage 2 or 3 in 6-well culture dishes with the Dnmt3b and GFP lentivirus.

### Statistical analysis

All data are presented as the mean ± standard deviation of at least three independent experiments. Significant intergroup differences were determined by one-way analysis of variance (ANOVA) followed by a Bonferroni *post hoc* multiple comparison test and Student’s t-test for two-component comparison after the normal distribution was confirmed. These differences were considered statistically significant when *P* < *0*.*05* (*P < *0*.*05*, **P < *0*.*001*).

## Supplementary information

Supplementary information
