# Supplementary material for: Effects of a hypomagnetic field on DNA methylation during the differentiation of embryonic stem cells
Source: Sci Rep. 2019 Feb 4;9:1333. doi: 10.1038/s41598-018-37372-2 (PMC6361932; doi:10.1038/s41598-018-37372-2)
Supplement: Supplementary file 1 — Supplementary information [file 41598_2018_37372_MOESM1_ESM.pdf]

SUPPLEMENTARY INFORMATION

Effects of a hypomagnetic field on DNA methylation during the differentiation of embryonic stem cells

Soonbong Baek [1], Hwan Choi[1], Hanseul Park[1], Byunguk Cho[1], Siyoung Kim[1] Jongpil Kim[1,2*]

[1]Department of Biomedical Engineering, Dongguk University, Seoul, 100-715, South Korea,

[2]Department of Chemistry, Dongguk University, Seoul, 100-715, South Korea,

Supplementary Figures

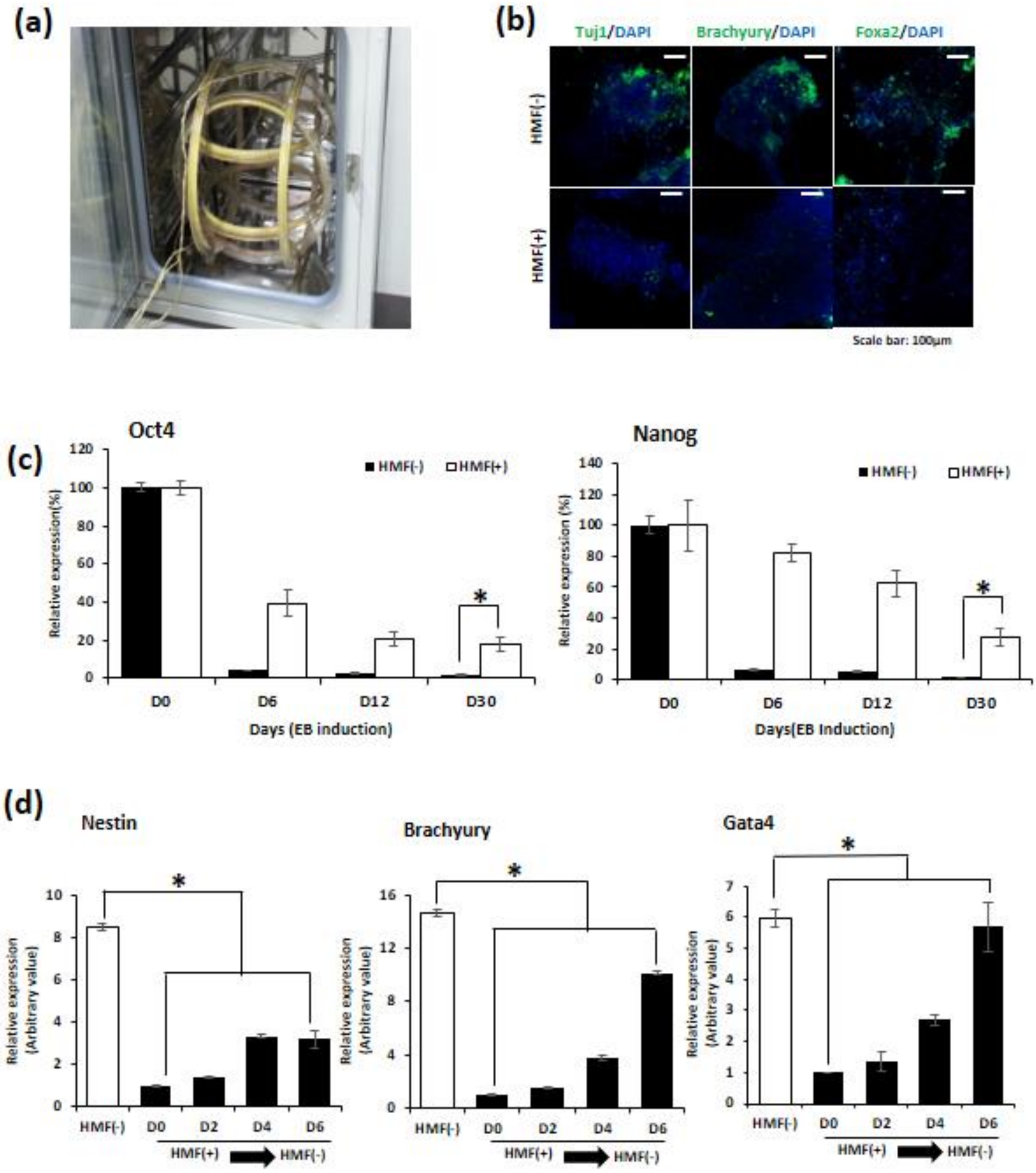

Supplementary Figure 1. Three-axis Helmholtz coil for HMF exposure. (a) Helmholtz coils were used to generate a uniform magnetic field. Three-axis coils are standard Helmholtz coils with varying dimensions that generate a single-axis, two-axis, or three-axis magnetic field. A 3D EMF generator was used to generate reverse magnetic fields to maintain a magnetic-field-free space by adjusting the voltage across the coil with a power supply. mESC culture dishes were placed at the center of the coils in a CO_2 incubator. (b) Representative immunofluorescence images of EBs for three germ layers (Tuj1, Brachyury and Foxa2) at Day 30. Experiments were performed in triplicate. (c) qRT-PCR analysis for Oct4 and Nanog mRNA expression in mESC differentiation under normal and HMF conditions at different time point, normalized to GAPDH. Data represent the mean ± SEM. Student's t-test*, *$*P < 0.05$ *(n=3).* (d) qRT-PCR analysis for marker of three germ layers (Nestin, Brachyury and Gata4) in mESC differentiation after HMF withdrawal. (HMF(-): differentiated mESC under normal conditions at day 6, D0, D2, D4, D6: days of HMF withdrawal of from differentiated mESC under HMF conditions at day 6). Data represent the mean ± SEM. Anova test*,* $*P < 0.05$ *(n=3).*

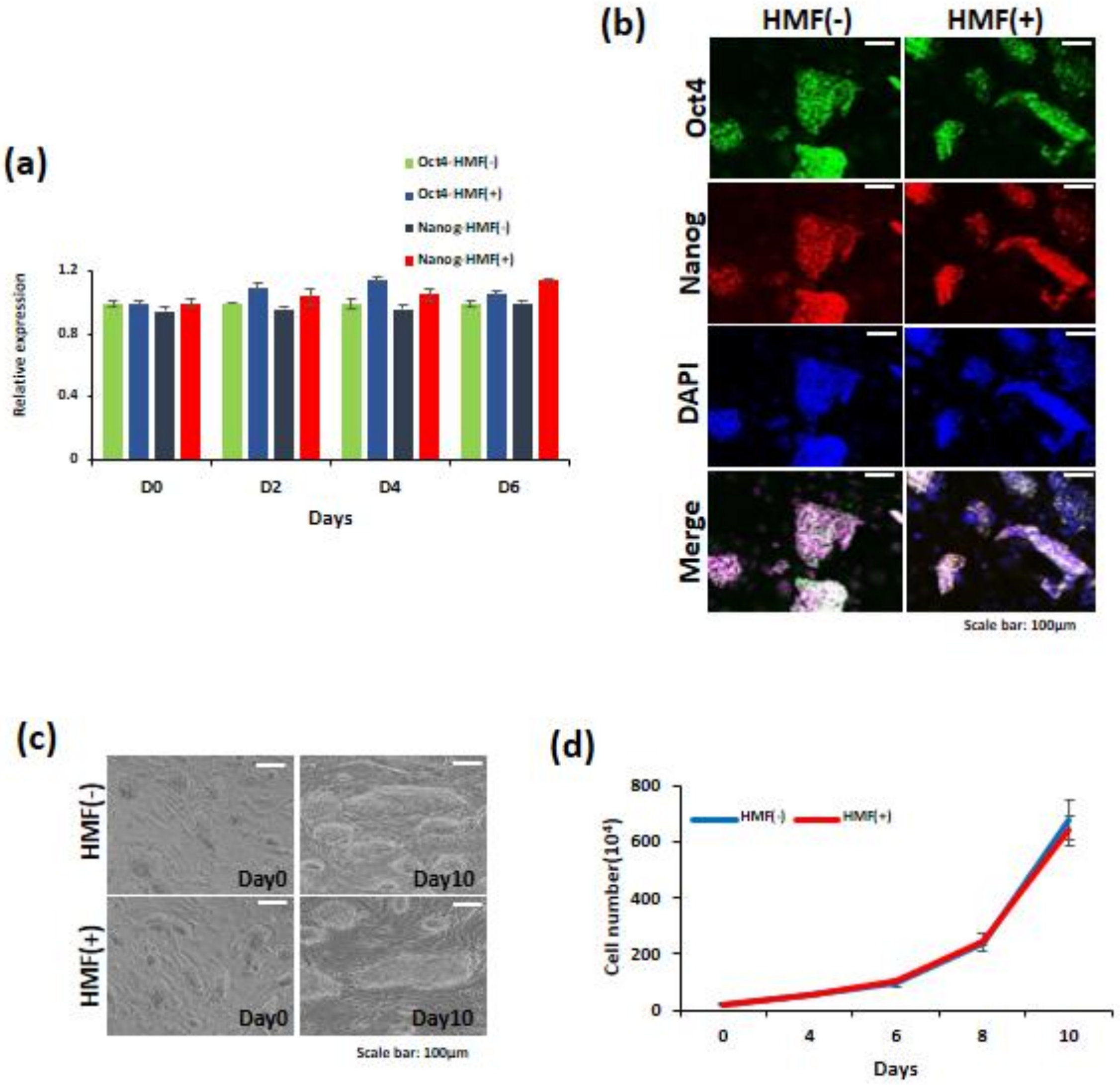

Supplementary Figure 2. Maintenance of pluripotent gene expression in HMF-treated mESCs. (a) Time course of Oct4 and Nanog mRNA expression in mESCs under normal and HMF conditions, normalized to GAPDH. (b) Immunofluorescence staining of the pluripotency markers Oct4 and Nanog in mESCs after 3 days under Normal (HMF(-)) and HMF(+) condition. Experiments were performed in triplicate. (c) Images show typical mESCs morphologies after 10 days under Normal (HMF(-)) and HMF(+) condition. Experiment were performed in triplicate. (d) Growth curves of mESCs under Normal (HMF(-)) and HMF(+) condition. Experiments were performed in triplicate.

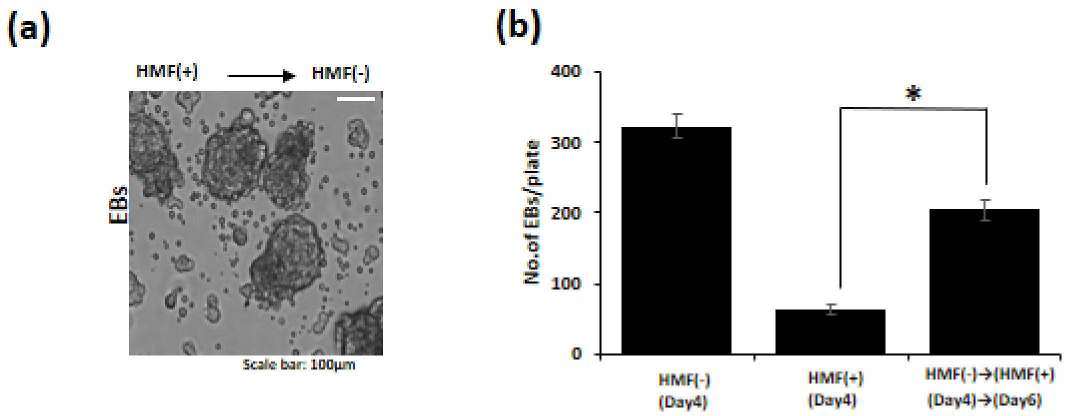

Supplementary Figure 3. mESC differentiation under HMF conditions. (a) Representative image of EBs after withdrawal of HMF (4 days at normal condition after 4 days under HMF condition). Experiments were performed in triplicate. (B) Number of EBs formed from mESCs under normal (HMF(-)), HMF(+) and withdrawal of HMF (4 days at normal condition after 4 days under HMF condition). Experiments were performed in triplicate.

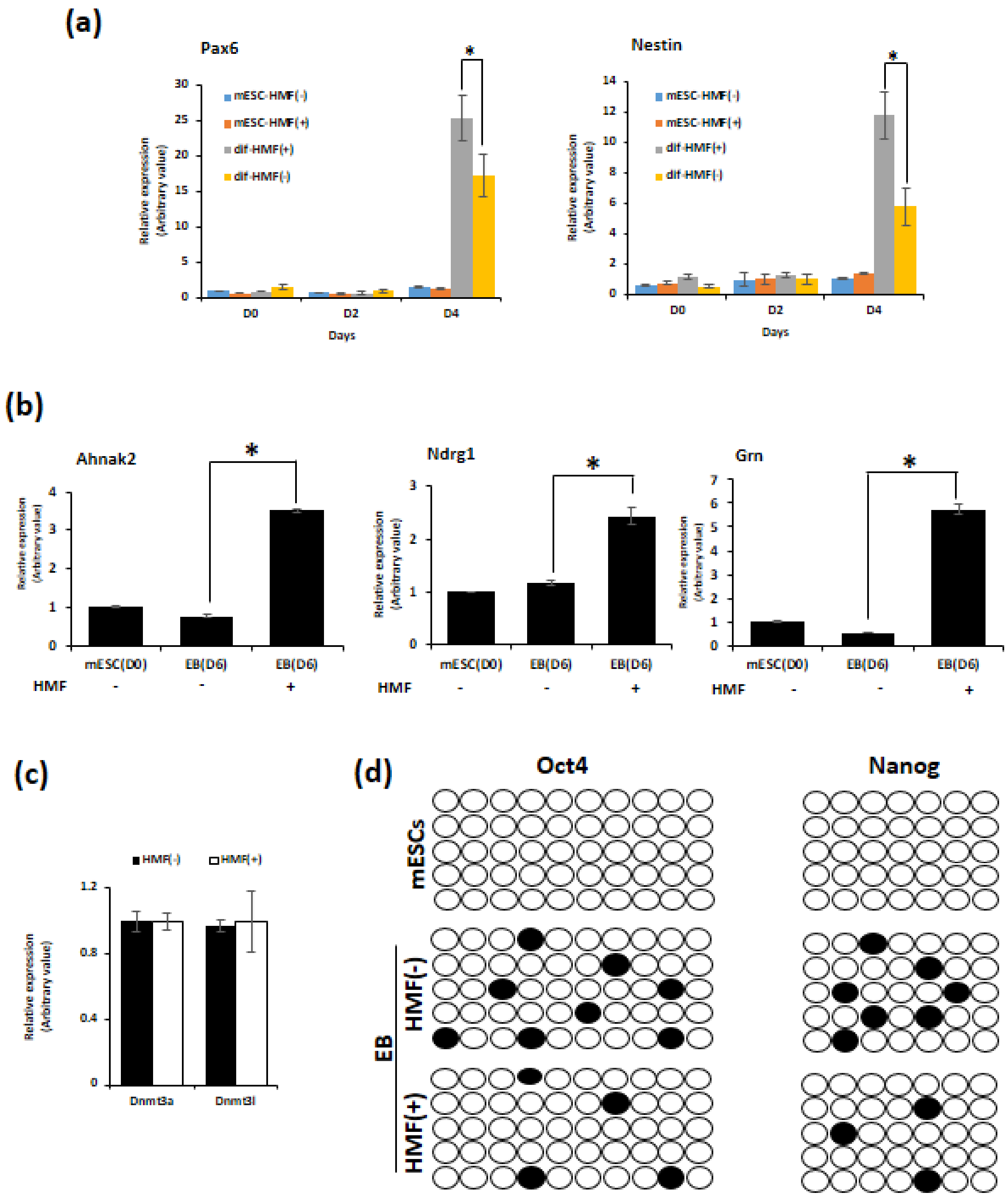

(a)
Pax6
mESC-HMF(-)
mESC-HMF(+)
dif-HMF(+)
dif-HMF(-)
Relative expression (Arbitrary value)
30
25
20
15
10
5
0
D0
D2
D4
Days
*
Nestin
14
12
10
8
6
4
2
0
(b)
Ahnak2
Ndrg1
Grn
mESC(D0)
EB(D6)
EB(D6)
HMF
-
-
+
(c)
HMF(-)
HMF(+)
1.2
0.8
0.4
0
Dnmt3a
Dnmt3l
(d)
Oct4
Nanog
mESCs
EB
HMF(-)
HMF(+)

Figure 4. Gene expression analysis of HMF conditions in mESC differentiation. (a) RT-PCR analysis for NSC maker, Pax6 and Nestin, of differentiated mESCs under Normal (HMF(-)) and HMF(+) condition, normalized to GAPDH. Data represent the mean ± SEM. Student's t-test, $*P < 0.05$ (n=3). (b) qRT-PCR analysis of development related genes, Ahnak2, Ndrg1 and Grn under normal (HMF(-)) and HMF(+) condition, normalized to GAPDH. Data represent the mean ± SEM. Student's t-test, $*P < 0.05$ (n=3). (c) mRNA expression of Dnmt3 family (Dnmt3a,b and l) under Normal (HMF(-)) and HMF(+) condition, normalized to GAPDH. Experiments were performed in triplicate. (d) Bisulfite sequencing of Oct4 and Nanog promoter in mESC and EB differentiated from mESCs under normal (HMF(-)) and HMF(+) condition.

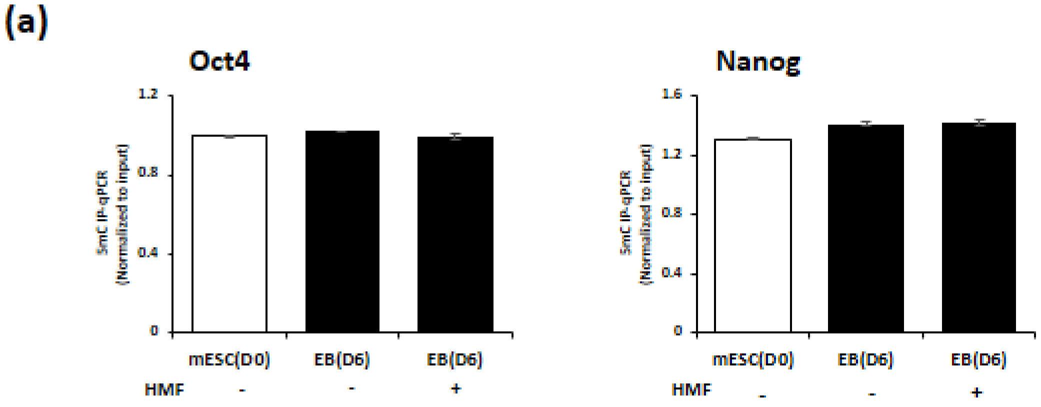

Figure 5. DNA methylation of mESCs under HMF condition. (a) IP-qRT-PCR for 5mC at Oct4 and Nanog promoter locus at 6 days after HMF treatment. Experiments were performed in triplicate.

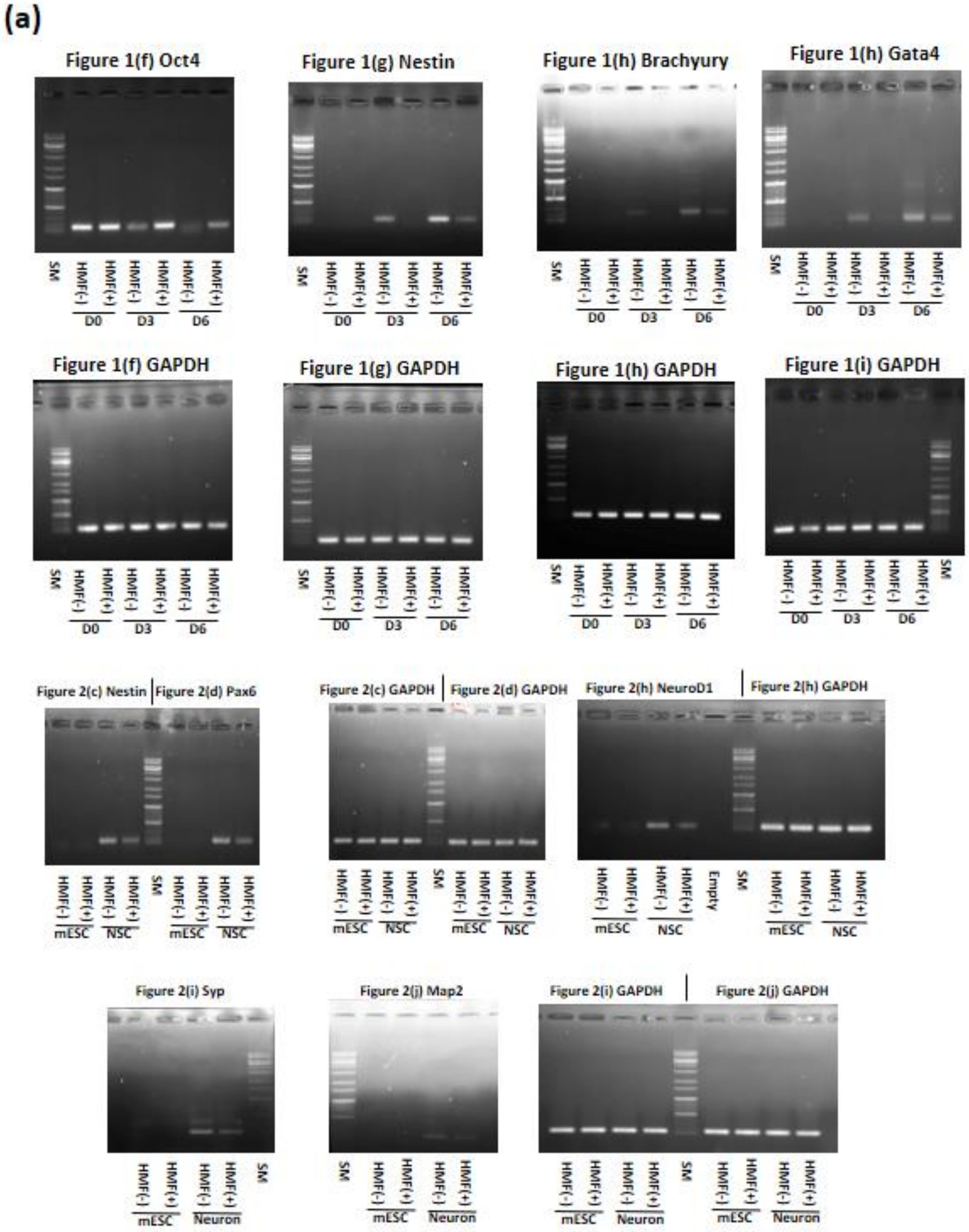

Figure 6. (a) The full-length RT-PCR bands corresponding to Figure 1f, g, h, 2c, 2d, 2h, 2i and 2j. SM: Size marker

(a)

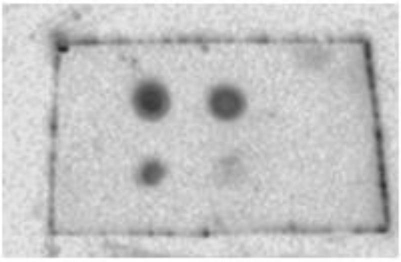

(b)

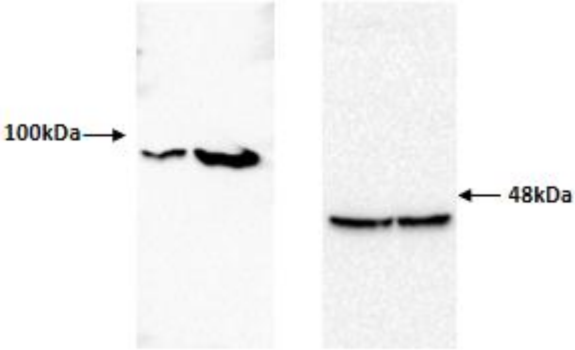

Figure 7. (a) Full-length dot blots for Figure 3e. (b) Full-length Western blot corresponding to the blot shown Figure 4a.

Supplementary Tables

Table 1. Upregulated gene in mESCs differentiation under HMF condition

Accession No.	Fold difference	Gene symbol	Gene title
BC138468	11.1	Ahnak2	Ahnak Nucleoprotein2
NM_008681	5.4	Ndrg1	N-Myc Downstream Regulated 1
NM_010421	4.7	Hexa	Hexoaminidase Subunit Alpha
NM_022032	3.8	Perp	PERP, TP53 Apoptosis Effector
NM_008175	4.5	Grn	Granulin precursor
NM_011930	2.8	Clcn7	Chloride Voltage-Gated Channel7

Table 2. Downregulated gene in mESCs differentiation under HMF condition

Accession No.	Fold difference	Gene symbol	Gene title
NM_001003961	-2.8	Dnmt3b	DNA Methyltransferase 3 Beta
NM_175485	-2.4	Prtg	Protogenin
NM_009830	-2.2	Ccne2	Cyclin E2
NM_145321	-2.2	Camkv	CaM kinase Like Vesicle Associated
NM_146909	-2.1	Olfr51	Olfactory Receptor Family 1 Subfamily
NM_009270	-2.1	Sqle	Squalene Epoxidase

Table 3. Positive cells for markers of three germ layers

Positive cell:		Tuj1/DAPI(%)	Brachyury/DAPI(%)	Foxa2/DAPI(%)
HMF(-)	#1	74/254(29.1)	32/264(12.1)	65/297(21.9)
	#2	82/321(25.5)	21/257(8.1)	75/301(24.9)
	#3	101/421(24.0)	13/286(4.5)	60/322(18.6)
HMF(+)	#1	78/265(29.4)	11/254(4.3)	54/264(20.4)
	#2	86/231(37.2)	23/256(8.9)	67/351(19.0)
	#3	66/321(20.5)	18/301(5.9)	77/275(28.0)
HMF(+)/Dnmt3b	#1	67/267(25.0)	35/562(6.2)	67/369(18.1)
	#2	88/301(29.2)	11/288(3.8)	55/255(21.5)
	#3	77/401(19.2)	21/305(6.8)	67/311(21.5)

Table 4. Primer information

Gene	Forward	Reverse
Pax6	tcagcttggtggtgtctttg	gcacctggacttttgcatct
Brachyury	tggaccccaatgccatgtac	gcgagtctgggtggatgtag
Gata4	ctgcagctggaaccacctc	ccagtagtccccgggaaaga
Nestin	agcagaaccagctgctcagt	cacctcagcctcgtgctt
NeuN	ccaagggttttgggtttgta	cccaggcttcttattggtca
Syp	aaagggggcactaccaagat	cattggccctttgttgttct
NeuroD1	tttgaaagccccctaactga	tgcagggtagtgcatggtaa
Map2	atgaaggaaaggcaccacac	gccttcctcctcctctctgt
Oct4	aacagtttgccaagctgctg	gcttcctccacccacttctc
Nanog	atgcctgcagtttttcatcc	gagcttttgtttgggactgg
Esrrb	caagagaaccattcaaggcaaca	catccccactttgaggcattt
GAPDH	tgcgacttcaacagcaactc	atgtaggccatgaggtccac
Dnmt3a	agcagaccaacatcgaatcc	cgtttccgtttgctgatgta
Dnmt3b	agtttccggctaccaggtct	accctcctgatctccatcct
Dnmt3l	cgacagctctagccctgatg	tcatctctctgcctgtcgga
Oct4-promoter	gctgggaattgaactccgga	ttccattcacagctgcgact
Nanog-promoter	taagctttccctccctccca	tcaagcctcctaccctaccc
Esrrb-promoter	gggcttttcactcgtggaga	cagctctcctggtttgaggg
